# Glaucoma in high myopia and parapapillary delta zone

**DOI:** 10.1371/journal.pone.0175120

**Published:** 2017-04-05

**Authors:** Jost B. Jonas, Pascal Weber, Natsuko Nagaoka, Kyoko Ohno-Matsui

**Affiliations:** 1 Department of Ophthalmology, Medical Faculty Mannheim of the Ruprecht-Karls-University of Heidelberg, Mannheim, Germany; 2 Department of Ophthalmology and Visual Science, Tokyo Medical and Dental University, Tokyo, Japan; Bascom Palmer Eye Institute, UNITED STATES

## Abstract

**Purpose:**

To examine the prevalence of glaucomatous optic neuropathy (GON) in a medium myopic to highly myopic group of patients and its association with parapapillary gamma zone and parapapillary delta zone.

**Methods:**

The retrospective observational hospital-based study included patients who had attended the Tokyo High Myopia Clinics within January 2012 and December 2012 and for whom fundus photographs were available. GON was defined based on the appearance of the optic nerve head on the fundus photographs.

**Results:**

The study included 519 eyes (262 individuals) with a mean age of 62.0±14.3 years (range:13–89 years) and mean axial length of 29.5±2.2 mm (range:23.2–35.3mm). GON was present in 141 (27.2%; 95% confidence intervals (CI): 23.3, 31.0%) eyes. Prevalence of GON increased from 12.2% (1.7, 22.7) in eyes with an axial length of <26.5mm to 28.5% (24.4, 32.5) in eyes with an axial length of ≥26.5mm, to 32.6% (27.9, 37.2) in eyes with an axial length of ≥28mm, to 36.0% (30.5, 41.4) in eyes with an axial length of ≥29mm, and GON prevalence increased to 42.1% (35.5, 48.8) in eyes with an axial length of ≥30mm. In multivariate analysis, higher GON prevalence was associated (Nagelkerke r^2^: 0.28) with larger parapapillary delta zone diameter (*P*<0.001; odds ratio (OR):1.86;95%CI:1.33,2.61), longer axial length (*P*<0.001;OR:1.45;95%CI:1.26,1.67) and older age (*P* = 0.01;OR:1.03;95%CI:1.01,1.05). If parapapillary delta zone width was replaced by the vertical disc diameter, higher GON prevalence was associated (r^2^:0.24) with larger vertical optic disc diameter (*P* = 0.04;OR:1.70;95%CI:1.03,2.81), after adjusting for longer axial length (*P*<0.001;OR:1.44;95%CI:1.26,1.64) and older age (*P*<0.001;OR:1.04;95%CI:1.02,1.06).

**Conclusions:**

Axial elongation associated increase in GON prevalence (mean: 28.1% in a medium to highly myopic study population) was associated with parapapillary delta zone as surrogate for an elongated peripapillary scleral flange and with larger optic disc size.

## Introduction

Glaucomatous optic neuropathy (GON) and maculopathies such as myopic maculopathy belong to the most common causes of irreversible visual loss worldwide [1,2]. Previous hospital-based studies and population-based investigations have shown that the prevalence of GON was higher in highly myopic eyes as compared to emmetropic eyes [3–9]. A recent study suggested that the increased frequency of GON in axially elongated eyes was associated with the size of the optic disc, i.e. GON was detected more frequently in highly myopic eyes with an enlarged optic disc than in highly myopic eyes with a normal-sized opic nerve head [10]. It was postulated that the enlargement of the optic disc including a stretching and thinning of the lamina cribrosa might have been one of the predisposing factors for the increased susceptibility for GON [10]. The latter study did not address the changes in the parapapillary region which were associated with high myopia, namely parapapillary gamma zone and parapapillary delta zone [11,12]. In particular parapapillary delta zone as the equivalent of the peripapillary scleral flange may however be an additional factor for an increased susceptibility for GON in highly myopic eyes [13,14]. The scleral flange as an extension of the posterior sclera is the biomechanical anchor of the lamina cribrosa. We therefore conducted this study to examine the occurrence of GON in a group of myopic patients and to assess whether parapapillary gamma zone and parapapillary delta zone were associated with the prevalence of GON.

## Methods

The retrospective observational hospital-based investigation included patients who attended the High Myopia Clinic of the Tokyo Medical and Dental University between January 2012 and December 2012. The study was approved by the Ethics Committee of the Tokyo Medical and Dental University. The procedures performed were in agreement with the tenets of the Declaration of Helsinki. Since it was a retrospective patient recruitment, since the character of the examinations was observational and non-invasive, and since the data had been obtained during routinely taken care of the patients, the Ethics Committee waived the necessity of obtaining an informed consent by the study participants. Inclusion criterion was the availability of fundus photographs of sufficient quality to allow the assessment of the optic disc and macula. Patients with marked opacities optic media were thus excluded.

The study design included a series of ophthalmological examinations such as refractometry, slit lamp based anterior segment biomicroscopy, tonometry, measurement of axial length (A-scan ultrasonography; Ultrascan, Alcon, Fort Worth, TX, USA), fundus examination under medical mydriasis, and color fundus photography (fundus camera: Topcon TRC 50DX; Topcon, Tokyo, Japan). For some eyes, optic disc photographs taken under standardized stereoscopic conditions were available, while for most eyes at least two disc photographs were taken and evaluated. Looking at both photographs of the optic disc (taken either under standardized stereoscopic conditions or under non-standardized conditions), the three-dimensional contour of the optic cup in delineation of the neuroretinal rim and the disc border was assessed.

Using the fundus photographs, we measured the horizontal, vertical, minimal and maximal optic disc diameter, the horizontal and maximal diameter of the parapapillary beta zone, gamma zone and delta zone, the distance between the most superior point of the temporal superior vascular arcade and the most inferior point of the of the temporal inferior vascular arcade, separated for the arteries and veins, the angle between the temporal vascular arcade and the optic disc (so called angle kappa), the number, location, maximal horizontal and maximal vertical diameter of chorioretinal atrophic lesions, the distance between the fovea and the outer border of gamma zone (i.e., the border of gamma zone in direction to the fovea), the disc-fovea distance. and the disc-fovea angle. All length measurements were corrected for their dependence on the magnification of fundus images using the method of Littmann–Bennett [15,16].

**Fig 1:**
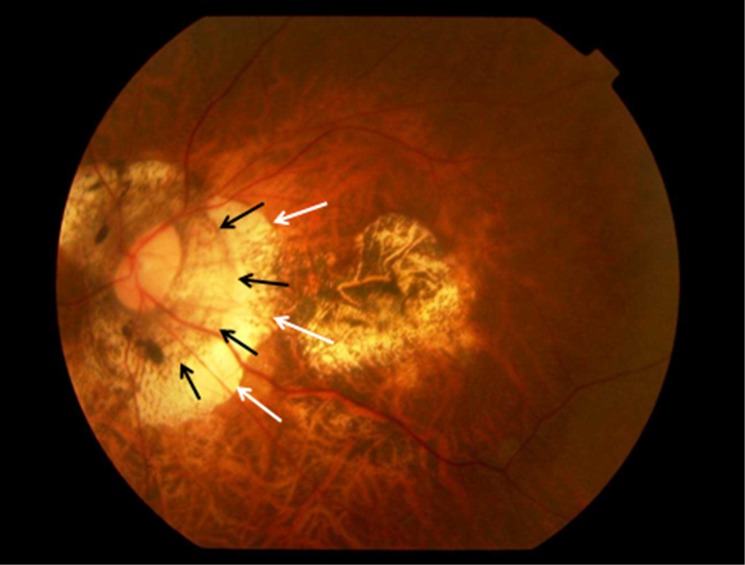
Fundus photograph of a highly myopic eye with parapapillary delta zone (black arrows) and gamma zone (white arrows).

**Fig 2:**
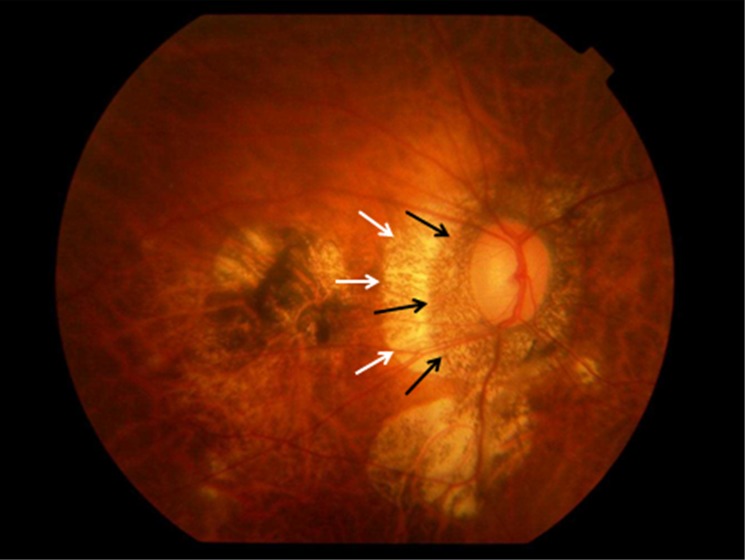
Fundus photograph of a highly myopic eye with parapapillary delta zone (black arrows) and gamma zone (white arrows).

Parapapillary gamma region and parapapillary delta region were defined as whitish areas at the temporal optic disc border without underlying choriocapillaris, without middle-sized choroidal vessels and without signs of retinal pigment epithelium (Figs 1 and 2) [11,12]. Delta zone, if detected, was located at the optic disc border (marked by the peripapillary ring), followed by gamma zone in direction to the fovea. The border between gamma zone and delta zone was a demarcation line which ran more or less parallel to the optic disc border and which could be located closely to the peripapillary arterial circle of Zinn-Haller. Beta zone was characterized by a location peripheral to gamma zone and showed whitish underground with large and medium-sized choroidal vessels (Figs 1 and 2) [17].

**Fig 3:**
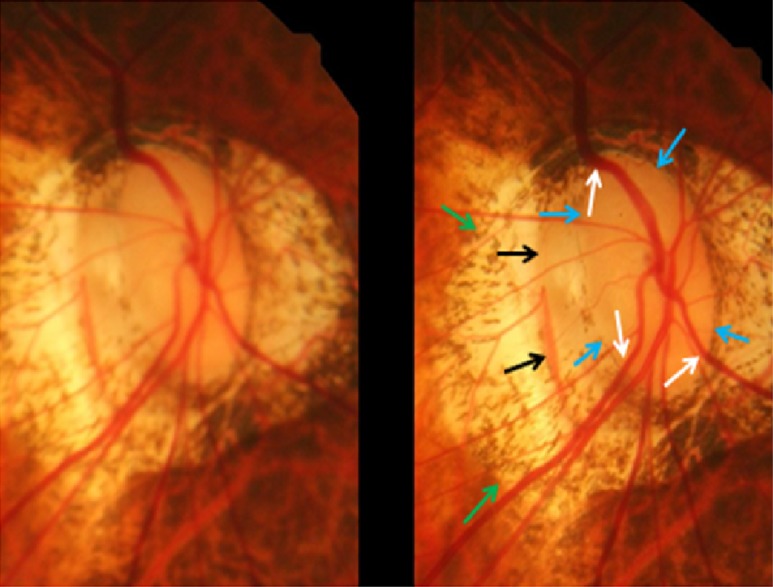
Stereoscopic photograph of a glaucomatous optic nerve head in a highly myopic eye with parapapillary gamma zone (green arrows), parapapillary delta zone (black arrows), and retinal vessel kinking close to the optic disc border (white arrows); blue arrows: optic disc border (peripapillary ring).

**Fig 4:**
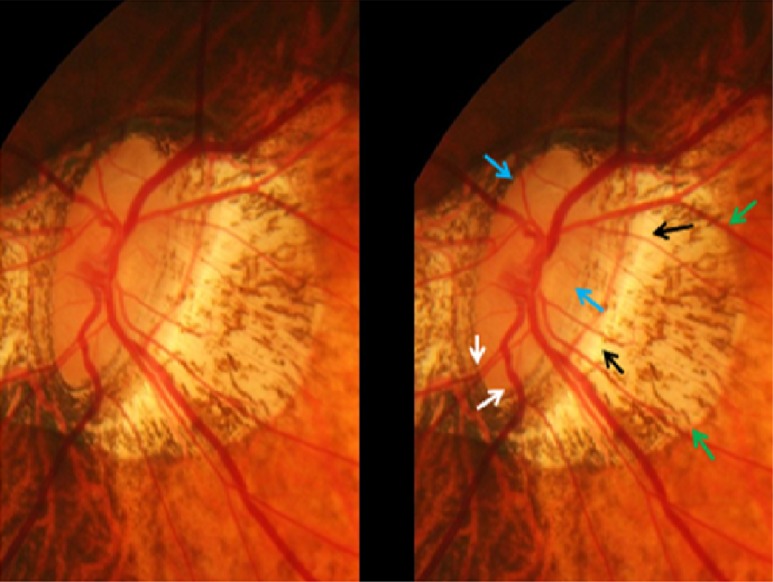
Stereoscopic photograph of a glaucomatous optic nerve head in a highly myopic eye with parapapillary gamma zone (green arrows), parapapillary delta zone (black arrows), and retinal vessel kinking close to the optic disc border (white arrows); blue arrows: optic disc border (peripapillary ring).

**Fig 5:**
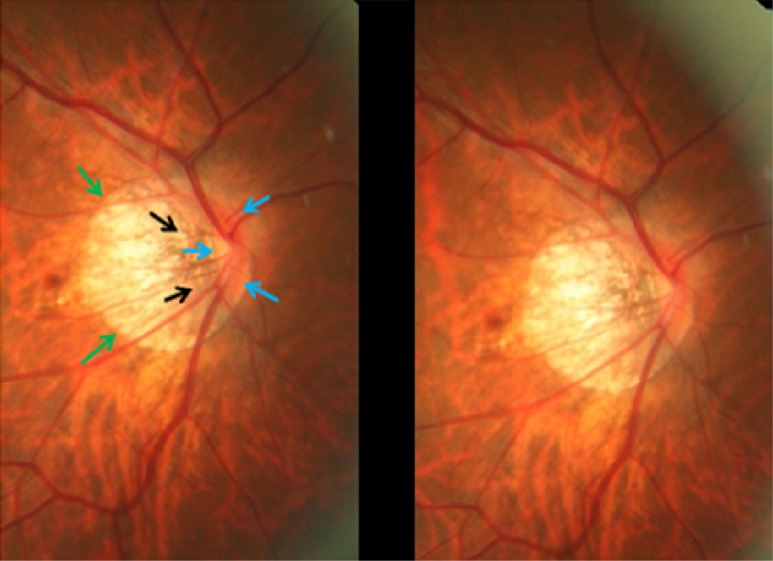
Stereoscopic photograph of an optic nerve head in a highly myopic eye without glaucomatous optic nerve damage, with parapapillary gamma zone (green arrows) and parapapillary delta zone (black arrows); blue arrows: optic disc border (peripapillary ring). The neuroretinal rim at the inferior disc pole and superior pole is markedly wider than in the temporal disc region in this optic disc with pronounced rotation around the vertical axis and a slight rotation (about 15°) around the sagittal axis.

GON was defined by the ophthalmoscopical appearance of the optic nerve head. The main criterion was the shape of the neuroretinal rim clearly showing a notch located in the inferior or superior disc region and representing a local, complete or almost complete, loss of neuroretinal rim at that point, or in a more advanced stage of GON, showing an extension of the optic cup (corresponding to an almost loss of neuroretinal rim) to the optic disc border in the inferior region, nasal region or superior region of the optic nerve head (Figs 3–5). The visibility of the retinal nerve fiber, the diameter of the retinal arteries, and the parapapillary alpha zone and beta zones were not taken into account [18].

In the statistical analysis (using SPSS version 22.0; IBM-SPSS, Chicago, IL, USA), we first determined the prevalence of GON and the mean and standard deviations of the main parameters in the glaucomatous group and in the non-glaucomatous separately. Additionally, we performed a univariate binary regression analysis with the presence of GON as dependent variable and the other parameters as independent variables. We finally carried out a multivariate regression analysis, with the presence of GON as the dependent variable and as independent variables all those parameters which were significantly associated with the presence of GON in the univariate analysis. We then dropped step by step those parameters which were no longer significantly associated with the presence of GON. We calculated the odds ratios (OR) and their 95% confidence intervals (CI). All *P*-values were two-sided and considered to be statistically significant if their value was lower than 0.05.

## Results

**Fig 6:**
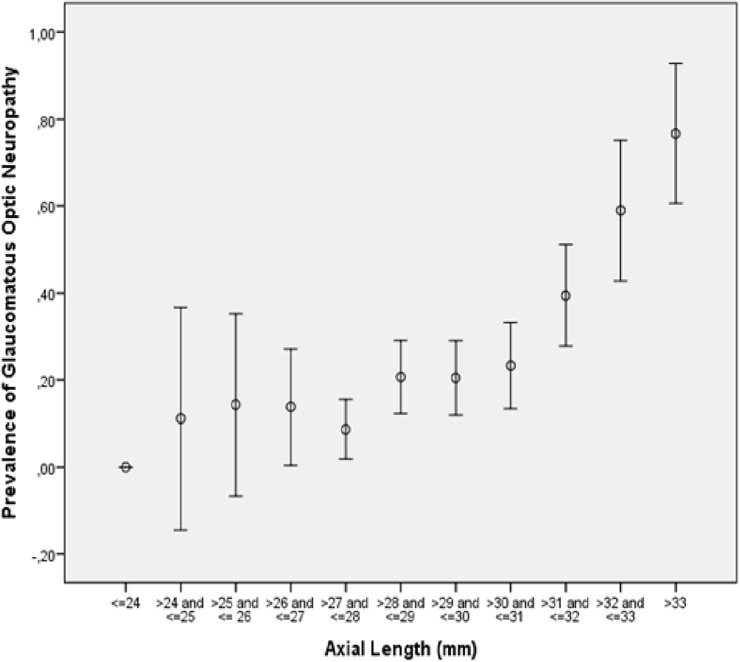
Graph Showing the Distribution of the Prevalence of Glaucomatous Optic Neuropathy and Axial Length.

The study included 519 eyes (262 individuals) with a mean age of 62.0 ± 14.3 years (range: 13–89 years) and a mean axial length of 29.5 ± 2.2 mm (range: 23.2–35.3mm). GON was present in 164 (28.1%; 95%CI: 24.4, 31.7) eyes. In the group of eyes with an axial length of <26.5mm, prevalence of GON was 12.2% (1.7, 22.7). Prevalence of GON increased to 28.5% (24.4, 32.5) in eyes with an axial length of ≥26.5mm, to 32.6% (27.9, 37.2) in eyes with an axial length of ≥28mm, to 36.0% (30.5, 41.4) in eyes with an axial length of ≥29mm, and GON prevalence increased to 42.1% (35.5, 48.8) in eyes with an axial length of ≥30mm (Fig 6).

The glaucomatous group differed significantly (univariate analysis) from the non-glaucomatous group by older age (*P*<0.001), longer axial length (*P*<0.001), larger parapapillary beta zone (*P* = 0.02), larger horizontal diameter (*P*<0.001) and larger vertical diameter (*P*<0.001) of parapapillary gamma zone and delta zone, shorter distance from the optic disc to the outer border of parapapillary gamma zone (*P* = 0.004), a longer disc-fovea distance (*P*<0.001), a larger horizontal diameter (*P*<0.001) and larger vertical diameter (*P*<0.001) of the largest chorioretinal lesion, higher count of chorioretinal lesions (*P*<0.001), and longer vertical diameter (*P*<0.001), longer maximal diameter (*P*<0.001), longer horizontal diameter (*P*<0.001) and longer minimal diameter (*P*<0.001) of the optic disc (Tables 1 and 2) (Fig 6). Both groups did not differ significantly in the vertical distance of the temporal arterial arcades (*P* = 0.13), angle kappa between the temporal superior arterial arcade and the temporal inferior arterial arcade (*P* = 0.28), the disc-fovea angle with the horizontal (*P* = 0.09), the angle between the disc-fovea line and the maximal optic disc diameter line (*P* = 0.48), the ratio of the vertical optic disc diameter to the horizontal optic disc diameter (*P* = 0.22), the ratio of the maximal optic disc diameter to minimal optic disc diameter (*P* = 0.96), and the angle between the maximal optic disc diameter line and the horizontal line (*P* = 0.13) (Table 2).

**Table 1:** Prevalence of Glaucomatous Optic Neuropathy Stratified by Axial Length.

Axial Length (mm)	n	Prevalence (%) (95% Confidence Intervals)
≤24	5	0.00
>24 and ≤25	9	11.1 (0, 37)
>25 and ≤26	14	14.3 (0, 35)
>26 and ≤27	29	14.3 (0, 27)
>27 and ≤28	69	8.7 (1.9, 15.5)
>28 and ≤29	92	20.7 (12.2, 29.1)
>29 and ≤30	88	20.5 (11.9, 29.1)
>30 and ≤31	73	23.3 (13.4, 33.2)
>31 and ≤32	71	39.4 (27.8, 51.1)
>32 and ≤33	39	59.0 (42.8, 75.1)
>33	30	76.7 (60.6, 92.7)

**Table 2:** Morphometric Parameters of the Fundus in the Glaucomatous Group Versus Non-Glaucomatous Group.

Parameter	Glaucomatous Group	Non-Glaucomatous Group	*P*-Value	Odds Ratio (OR)	95% Confidence Interval of OR
Age (Years)	66.3 ± 12.5	61.1 ± 14.8	<0.001	1.03	1.01, 1.04
Axial Length (mm)	30.8 ± 2.2	29.1 ± 2.0	<0.001	1.52	1.37, 169
Vertical Diameter of Temporal Arterial Arcade (mm)	7.61 ± 1.8	7.98 ±1.67	0.13	0.90	0.79., 1.03
Angle Kappa between temporal Superior Arterial Arcade and Temporal Inferior Arterial Arcade (°)	90.2 ± 20.1	92.1 ± 16.0	0.28	0.99	0.99, 1.00
Parapapillary Beta Zone Area (mm^2^)	8.84 ± 8.00	6.40 ± 12.90	0.02	1.02	0.99, 1.04
Parapapillary Gamma Zone, Horizontal Diameter (mm)	7.93 ± 2.49	6.06 ± 1.74	<0.001	1.53	1.36, 1.72
Parapapillary Gamma Zone, Vertical Diameter (mm)	8.44 ± 3.16	6.06 ± 2.07	<0.001	1.43	1.30,1.58
Parapapillary Delta Zone, Horizontal Diameter (mm)	3.20 ± 0.91	2.53 ± 0.58	<0.001	2.87	2.12, 3.89
Parapapillary Delta Zone, Vertical Diameter (mm)	3.68 ± 1.34	2.79 ± 0.86	<0.001	2.18	1.73, 2.75
Gamma Zone Combined with Delta Zone, Horizontal Diameter (mm)	7.93 ± 2.49	6.06 ± 1.74	<0.001	1.53,	1.36, 1.72
Gamma Zone Combined with Delta Zone, Vertical Diameter (mm)	8.44 ± 3.16	6.07 ± 2.07	<0.001	1.43	1.30, 1.58
Distance Fovea–Outer Border of Gamma Zone (mm)	2.72 ± 0.98	2.99 ±0.73	0.004	0.67	0.52, 0.85
Disc–Fovea Angle with Horizontal (°)	8.39 ± 5.25	9.24 ± 5.43	0.09	0.97	0.94, 1.01
Angle between Disc-Fovea Line and Maximal Optic Disc Diameter Line (°)	92.3 ± 30.2	90.3 ± 28.4	0.48	1.00	0.00, 1.01
Disc-Fovea Distance (mm)	5.90 ± 0.90	5.53 ± 0.73	<0.001	1.82	1.40, 2.35
Fundus Tessellation	2.89 ± 0.86	3.14 ± 0.81	0.001	0.69	0.55, 0.86
Horizontal Diameter of Largest Chorioretinal Lesion	3.65 ±3.80	1.82 ± 2.76	<0.001	1.20	1.13, 1.28
Vertical Diameter of Largest Chorioretinal Lesion	3.68 ± 3.80	1.87 ± 2.61	<0.001	1.20	1.13, 1.27
Count of Chorioretinal Lesions	0.96 ± 1.12	0.63 ± 1.01	0.001	1.33	1.13, 1.57
Optic Disc, Horizontal Diameter (mm)	1.74 ±0.49	1.49 ± 0.42	<0.001	3.34	2.13, 5.23
Optic Disc, Vertical Diameter (mm)	2.18 ± 0.57	1.87 ± 0.47	<0.001	3.23	2.18, 4.80
Optic Disc, Minimal Diameter (mm)	1.57 ± 0.45	1.34 ± 0.39	<0.001	3.58	2.21, 5.81
Optic Disc, Maximal Diameter (mm)	2.43 ± 0.54	2.13 ± 0.44	<0.001	3.57	2.34, 5.46
Optic Disc, Vertical Diameter / Horizontal Diameter	1.31 ± 0.35	1.31 ± 0.34	0.22	1.04	0.61, 1.77
Optic Disc, Maximal Diameter / Minimal Diameter	1.62 ± 0.39	1.67 ± 0.42	0.96	0.75	0.46, 1.21
Angle between Maximal Optic Disc Diameter Line and Horizontal Line (°)	85.9 ± 29.4	81.8 ± 29.4	0.13	1.01	1.00, 1.01

The binary multivariate regression analysis included the presence of GON as dependent variable and all those parameters as independent variables which were significantly associated with the presence of GON in the univariate analysis. Due to collinearity, we chose the minimal disc diameter and maximal disc diameter as parameters for optic disc size and dropped the horizontal disc diameter and the vertical disc diameter. Another reason for dropping the latter two parameters was the distortion of the image of the optic disc on two-dimensional optic disc photographs, caused by the rotation of the optic nerve head mainly around its vertical axis in axially elongated eyes [19]. In a step-wise manner, we dropped the horizontal diameter of gamma zone (*P* = 0.99), beta zone area (*P* = 0.68), the vertical diameter of delta zone (*P* = 0.97), the count of chorioretinal lesions (*P* = 0.62), the fovea-outer gamma zone border distance (*P* = 0.41), the vertical diameter of gamma zone (*P* = 0.48), the vertical diameter of the largest chorioretinal lesion (*P* = 0.37), the horizontal diameter of the largest chorioretinal lesion (*P* = 0.50), the disc-fovea distance (*P* = 0.42), the degree of fundus tessellation (*P* = 0.24), the maximal disc diameter (*P* = 0.34), and the minimal disc diameter (*P* = 0.21). In the final model, higher prevalence of GON was associated (Cox & Snell´s r^2^: 0.19; Nagelkerke r^2^: 0.28) with a larger width of parapapillary delta zone (*P*<0.001; OR: 1.86; 95%CI: 1.33, 2.61), longer axial length (*P*<0.001; OR: 1.45; 95%CI: 1.26, 1.67), and older age (*P* = 0.01; OR: 1.03; 95%CI: 1.01, 1.05) (Table 3). If delta zone width was dropped and the vertical disc diameter was added to the list of independent variables, a higher prevalence of GON was associated (Cox & Snell´s r^2^: 0.16; Nagelkerke´s r^2^: 0.24) with larger vertical optic disc diameter (*P* = 0.04; OR: 1.70; 95%CI: 1.03, 2.81), longer axial length (*P*<0.001; OR: 1.44; 95%CI: 1.26, 1.64), and older age (*P*<0.001; OR: 1.04; 95%CI: 1.02, 1.06) (Table 3).

**Table 3:** Associations (Multivariate Analysis) between the Prevalence of Glaucomatous Optic Neuropathy and Ocular and Systemic Parameters.

Parameter	*P*-Value	Odds Ratio (OR)	95% Confidence Interval of OR
Width of Parapapillary Delta Zone (mm)	<0.001	1.86	1.33, 2.61
Axial Length (mm)	<0.001	1.45	1.26, 1.67
Age (Years)	0.01	1.03	1.01, 1.05
After Replacing Delta Zone Width by Vertical Disc Diameter			
Axial Length (mm)	<0.001	1.44	1.26, 1.64
Age (Years)	<0.001	1.04	1.02, 1.06

## Discussion

In our medium myopic and highly myopic study population, the overall prevalence of GON was 28.1% (95%CI: 24.4, 31.7%), with a steep increase in prevalence from 12.2% in the group of eyes with an axial length of <26.5mm to 28.5% in the group of eyes with an axial length of ≥26.5mm, and to and 42.1% in the group of eyes with an axial length of ≥ 30mm (Fig 6). A higher prevalence of GON was significantly correlated with a larger parapapillary delta zone and longer axial length after adjusting for older age (Table 2). If parapapillary delta zone was replaced by a disc size parameter, prevalence of GON increased with larger vertical optic disc diameter (Table 2). The results suggest that the axial elongation associated increase in the prevalence of GON was associated with a profound stretching of the peripapillary scleral flange as histological equivalent of the parapapillary gamma zone. As a corollary, axial elongation associated increase in the prevalence of GON was correlated with a larger optic disc size after adjusting for older age.

The results of our study confirm previous studies on an increased prevalence of glaucoma in highly myopic eyes [3–9]. In the Beijing Eye Study on 4319 individuals, prevalence of GON was significantly (*P*<0.001) higher in eyes with a myopic refractive error of more than -6 diopters than in eyes with a myopic refractive error of less than -3 diopters [8]. In a hospital-based study on 172 patients with a mean axial length of 30.1 ± 2.3 mm (range: 24.7–39.1mm), Nagaoka and colleagues found that the prevalence of GON (overall: 28%) was 3.2 times higher (*P*<0.001) in large optic discs (>3.79 mm^2^) than in normal-sized discs or small discs (<1.51 mm^2^) after adjusting for older age. Interestingly, axial length was not significantly (*P* = 0.38) associated with glaucoma prevalence in that model [10].

The new finding of our study is that the increased prevalence of GON in the axially myopic eyes was correlated with the size of parapapillary delta zone. Delta zone has been defined at the region between the peripapillary ring (which is the extension of the optic nerve pia mater) and the merging line of the optic nerve dura mater with the posterior sclera [11,12,20]. It represents the peripapillary scleral flange as continuation of the inner half of the posterior sclera to the lamina cribrosa [11–13]. In non-axially elongated non-glaucomatous eyes, the thickness and length of the peripapillary scleral flange is about 0.5 mm, with its thickness being comparable with the thickness of the peripheral lamina cribrosa [21]. The peripapillary scleral flange forms the roof at the anterior end of the orbital cerebrospinal fluid space [13]. Since the peripapillary scleral flange connects the lamina cribrosa with the posterior sclera, it can be considered as the biomechanical anchor of the lamina cribrosa [14,22]. If the peripapillary scleral flange is compared with the pylon of a suspension bridge which would serve as a model for the lamina cribrosa, the tension in the pylon is the larger the shorter the pylon is. Transferred to the anatomy of the optic nerve, one may postulate that the thinner the peripapillary scleral flange is, the higher is the tension in the peripapillary scleral flange and in the lamina cribrosa. Since in highly axially elongated eyes, the peripapillary scleral flange (i.e. parapapillary delta zone) can be elongated by a factor of 10 and since the thickness of the peripapillary scleral flange can be reduced to 10% of its normal value, the association of a higher prevalence of GON with a larger parapapillary delta zone may be based on histologic changes accompanying parapapillary delta zone. It may also be of potential interest to assess the association between the trans-lamina cribrosa pressure difference and trans-lamina cribrosa gradient and the histologic particularities of the parapapillary region in highly myopic eyes [23].

Interestingly, the size of parapapillary gamma zone was not associated with the prevalence of GON after adjusting for axial length, age and size of parapapillary delta zone (Table 3). Gamma zone is defined as the Bruch´s membrane free region peripheral (i.e. in direction to the fovea) to the merging line of the optic nerve dura mater with the posterior sclera. The thickness of the sclera in gamma zone is usually at least double as thick as the thickness of the peripapillary scleral flange inside of delta zone. This finding together with the longer distance between the lamina cribrosa and gamma zone as compared to delta zone may explain, why gamma zone, in contrast to delta zone, was not related to an increased prevalence of GON in the myopic eyes.

The findings obtained in this study may be clinically helpful. If a highly myopic patient has a large delta zone and or a large optic disc, the risk for this patient to have glaucoma may be higher than for a highly myopic patient without delta zone and/or with a normal-sized optic disc. It may show the potential usefulness of differentiating between parapapillary gamma zone and parapapillary delta zone.

Limitations of our study should be taken into account. First, it was a hospital-based study so that features of the study population examined in a third-referral center for high myopia may not be representative for the general myopic or highly myopic population. The recruitment of the study participants may therefore have caused a bias. The study participants however attended the hospital and were included into the study based on their myopia while glaucoma was not a reason for attending the hospital which has been well-known for myopia. Also, the presence of glaucoma was not known for all participants affected by GON at the time of their first examination in the hospital. It may therefore not be likely, that the hospital-based recruitment of the study participants may have caused a major bias. Second, a major concern in this study may be that the diagnosis of GON was made solely via the photographic examination of the optic nerve head. Being observational and cross-sectional, without taking into account intraocular pressure, results of visual field examinations or findings obtained by optical coherence tomography, the distinction between optic discs with a normal optic nerve and eyes with GON may be doubted. Previous population-based studies from East Asia have shown however, that a single intraocular pressure measurement is within the normal range in the majority of patients with open-glaucoma, in particular in those glaucomatous patients with high myopia [24,25]. It suggested not to use intraocular pressure as criterion for the diagnosis of glaucoma. The reason why perimetric results were not considered for the diagnosis of GON was that visual field defects in highly myopic eyes can have a multitude of reasons besides glaucoma, including choroidal and retinal changes due to the myopic axial elongation. Due to the retrospective character of the study design, optical coherence tomographic images of the optic nerve head were not available. OCT images are however, often not very helpful in detecting and quantifying the amount of GON in highly myopic eyes. Third, for the diagnosis of GON as applied in our study, the neuroretinal rim had to have a clearly glaucoma-like appearance, either in the form of rim notches touching or almost touching the disc border or in the form of an advanced loss of neuroretinal rim with an optic cup extending to the disc border for a large sector of the optic nerve head. This definition of GON might have led to an underestimation of the prevalence of GON since early stages of glaucoma, before the development of clear neuroretinal rim notches, might have been considered to be non-glaucomatous. This weakness in the assessment of GON may serve to underline the results of the present study that the observed high prevalence of GON in highly myopic eyes might in reality have been even higher than reported. Fourth, some of the disc photographs were taken in a non-stereoscopic manner. There were however, usually at least two photographs for each optic disc available, so that the simultaneous observation of both optic disc images allowed some stereoscopic analysis.

In conclusion, about 30% of our medium myopic or highly myopic study population had GON, with a steep increase in the prevalence from 12.2% in the group of eyes with an axial length of <26.5mm to 28.5% in the group of eyes with an axial length of ≥26.5mm, and to and 42.1% in the group of eyes with an axial length of ≥ 30mm. Axial elongation associated increase in GON prevalence was associated with a marked stretching of the peripapillary scleral flange as histological equivalent of the ophthalmoscopical parapapillary delta zone, and as a corollary, with a larger optic disc size after adjusting for older age.
